# Factors influencing survival time of hemodialysis patients; time to event analysis using parametric models: a cohort study

**DOI:** 10.1186/s12882-019-1382-2

**Published:** 2019-06-11

**Authors:** Vahid Ebrahimi, Mohammad Hossein Khademian, Seyed Jalil Masoumi, Mohammad Reza Morvaridi, Shahrokh Ezzatzadegan Jahromi

**Affiliations:** 1 Shiraz Nephro-Urology Research Center, Shiraz University of Medical Sciences, Shiraz, Iran; 2 Colorectal Research Center, Shiraz University of Medical Sciences, Shiraz, Iran; 3 Department of Medical Surgical Nursing, School of Nursing and Midwifery, Shiraz University of Medical Sciences, Shiraz, Iran; 4 Gastroenterohepatology Research Center, Shiraz University of Medical Sciences, Shiraz, Iran; 5 School of Nutrition and Food Sciences, Shiraz University of Medical Sciences, Shiraz, Iran; 6 Nutrition and Food Sciences Research Center, Shiraz University of Medical Sciences, Shiraz, Iran; 7 Department of Internal Medicine, Nemazee Hospital, Shiraz, Iran

**Keywords:** Body mass index, Erythrocyte count, Leukocyte count, Renal dialysis, Serum albumin, Survival analysis, Ultrafiltration

## Abstract

**Background:**

Survival analysis of patients on maintenance hemodialysis (HD) has been the subject of many studies. No study has evaluated the effect of different factors on the survival time of these patients. In this study, by using parametric survival models, we aimed to find the factors affecting survival and discover the effect of them on the survival time.

**Methods:**

As a retrospective cohort study, we evaluated the data of 1408 HD patients. We considered the data of patients who had at least 3 months of HD and started HD from December 2011 to February 2016. The data were extracted from Shiraz University of Medical Sciences (SUMS) Special Diseases database. Primary event was death. We applied Cox-adjusted PH to find the variables with significant effect on risk of death. The effect of various parameters on the survival time was evaluated by a parametric survival model, the one found to have the best fit by Akaike Information Criterion (AIC).

**Results:**

Of 428 HD patients eligible for the analysis, 221 (52%) experienced death. With the mean ± SD age of 60 ± 16 years and BMI of 23 ± 4.6 Kg/m, they comprised of 250 men (58%). The median of the survival time (95% CI) was 624 days (550 to 716). The overall 1, 2, 3, and 4-year survival rates for the patients undergoing HD were 74, 42, 25, and 17%; respectively. By using AIC, AFT log-normal model was recognized as the best functional form of the survival time. Cox-adjusted PH results showed that the amount of ultrafiltration volume (UF) (HR = 1.146, *P* = 0.049), WBC count (HR = 1.039, *P* = 0.001), RBC count (HR = 0.817, *P* = 0.044), MCHC (HR = 0.887, P = 0.001), and serum albumin (HR = 0.616, *P* < 0.001) had significant effects on mortality. AFT log-normal model indicated that WBC (ETR = 0.982, *P* = 0.018), RBC (ETR = 1.131, *P* = 0.023), MCHC (ETR = 1.067, P = 0.001), and serum albumin (ETR = 1.232, 0.002) had significant influence on the survival time.

**Conclusion:**

Considering Cox and three parametric event-time models, the parametric AFT log-normal had the best efficiency in determining factors influencing HD patients survival. Resulting from this model, WBC and RBC count, MCHC and serum albumin are factors significantly affecting survival time of HD patients.

## Background

The number of patients with end-stage renal disease (ESRD) requiring renal replacement therapy (RRT) has increased over the last decades all over the world. Such rise in the population of ESRD patients was more prominent in the developed countries. Hemodialysis (HD), followed by kidney transplantation and peritoneal dialysis (PD), is the most widely used type of renal replacement therapy. One of the major concerns in the field of HD is the high mortality rate of these patients; therefore, the evaluation of the factors influencing survival of HD patients has been the subject of several studies.

Because there are HD patients who do not experience death and are alive at the end of follow-up time, the information relating to these patients are not taken into consideration for the analysis, i.e., being censored, in the traditional regression statistical methods. Thus, these traditional regression statistical methods, such as logistic or multiple linear regressions, are not appropriate for the survival analysis in HD patients. Consequently, survival analyses methods which consider censored events are more appropriate for analyzing variables influencing survival in patients undergoing HD [1, 2].

One of the most commonly utilized approaches for the survival analysis is multivariate semi-parametric Cox-adjusted proportional hazards (PH) model, in which it would be necessary to check the PH assumption of proportionality of hazards (i.e. constant hazard ratios over time) for each factor. According to the result of a systematic review, the assumption of proportionality has not been met in most of the studies where this type of survival analysis has been used [1–3].

Another type of the survival models are parametric models, in which the distribution of the survival time is specified [1]. Accelerated failure time (AFT) method is one of the parametric survival models as an alternative to the Cox-PH method. A variety of models could be defined for AFT model such as Weibull, log-normal, and log-logistic models [1, 2]. Whereas semi-parametric survival time methods only focus on the influence of factors on hazard of death, parametric survival models can also compute the distribution form of the survival time, i.e. AFT parametric methods express the multiplicative effect of factors on the time to death. In addition, parametric survival models have more power than semi-parametric Cox methods in finding factors associated with survival, provided their parametric form are correctly determined [2, 4].

In most situations, even if all the conditions for the Cox-PH method are met, parametric survival models may be superior to the Cox model; under certain circumstances, they even provide better estimates than the Cox-adjusted methods [5].

In medical literature, hazard ratio (HR) is more widely reported and is more familiar to the majority of the clinicians, while the relative change in the survival time, which is called event time ratio (ETR), is easier to comprehend [6]. ETR is the direct effect of some exposure and/or a factor on the survival time, i.e. survival time increases by a factor of the estimated ETR for each unit increment in that variable or factor (if that variable is quantitative) or for that category of the variable compared to the other (if that variable is qualitative). Since there are no studies using ETR for analyzing survival in HD population, we aimed to identify the factors that might be independently associated with survival in HD patients’ population by using parametric methods in order to achieve the standard estimation of the survival function and clinical relevant statistic.

## Methods

### Sampling population

The data in the current retrospective cohort survey was extracted from Shiraz University of Medical Sciences (SUMS) Specific Diseases Affairs electronic database. We included the information of the patients who started maintenance standard HD from December 2011 to February 2016, [*n* = 1014] in 32 Fars province HD centers in Iran.

The patients who survived 3 months of maintenance HD were brought in for the analysis. Exclusion criteria were deficit in data, switching to PD or kidney transplantation during the follow up period, and loss of follow-up because of being transferred to an HD center not covered by the database. The time interval from the very start (4 months after the first dialysis) to the end of the study was considered as censored time if the event of interest, i.e. death, did not occur in that interval.

### Demographic and clinical data of the study patients

Data of the initial 3 months on HD were not taken in for the analysis. The demographical characteristics of the HD patients including gender, age, body mass index (BMI), and the underlying cause of ESRD were considered. HD sessions-related indices which belong to the 4th month of starting HD were the average of ultrafiltration volumes and dialysis durations, as well as pre-dialysis weight, post-dialysis weight, pre-dialysis systolic blood pressure (SBP), diastolic blood pressure (DBP), and pulse rate were employed for the analysis as well.

The following variables which belong to the 4th month of starting HD were used in the analysis: total white blood cell count (WBC), red blood cell count (RBC), serum hemoglobin concentration (Hb), hematocrit (HCT), mean cell volume (MCV), mean cell hemoglobin concentration (MCH), mean corpuscular hemoglobin concentration (MCHC), serum concentrations of fasting blood sugar (FBS), pre-dialysis blood urea nitrogen (BUN), post-dialysis blood urea nitrogen (BUN), pre-dialysis serum creatinine, post-dialysis serum creatinine, calcium, phosphate, sodium, and potassium. Additionally, serum concentrations of uric acid, cholesterol, triglyceride, low density lipoprotein (LDL), high-density lipoprotein (HDL), ferritin, Iron, total iron binding capacity (TIBC), parathyroid hormone (PTH), total protein, albumin, and alkaline phosphatase (ALKP) were considered. Variables related to the HD sessions—such as the adequacy of HD—was measured by single-pool *Kt*/*V*_*urea*_ [7]. Lab samples were not centralized and were measured in HD centers laboratories. They were all obtained before dialysis sessions.

### Statistical analyses

In this study, survival analysis was applied in three ways: *non-parametric, semi-parametric*, and *parametric* methods. Survival and hazard estimates of the mortality of the HD patients were based on non-parametric Kaplan-Meier (KM) approach. Because non-parametric survival models do not adjust for covariates, we used semi-parametric models(i.e. Cox-adjusted PH model)and parametric survival models in order to find out the model that best fits our data [1, 2].

In point of fact, the main goal of the Cox-adjusted model is to simultaneously evaluate the effect of several factors on the probability of survival [1]. Time to the death was analyzed using the Cox-adjusted PH model, via Breslow method, to estimate the hazard of death. After checking PH assumption (via the goodness-of-fit testing method), the results were interpreted using hazard ratio (HR) as the averaged hazards of death.

In order to find the best functional form of the survival time, we constructed four types of parametric event-time models including PH Gompertz and AFT models (Weibull, lognormal, and log-logistic) [1, 2].

We used various methods to check the adequacy of the parametric survival models such as comparing Akaike’s information criteria (AIC) of the models, graphical comparison between non-parametric and parametric methods, and plot of the empirical estimates of the cumulative hazard function (based on the Kaplan-Meier (KM) survival estimates when Cox-Snell residuals are considered as the time variable) [1, 8, 9].

The Akaike information criterion (AIC) is an estimator designed for comparing appropriateness and relative quality of the fitted models and, therefore, is a mean for model selection. The model with the smallest AIC score was selected as the best [1]. The results of the optimal accelerated failure time (AFT) parametric model were also interpreted via event-time-ratio (ETR) statistics as the averaged increase in the survival time.

Selection of the variables was based on the clinical importance as well as the result of the univariate analysis, such that, variables with *p*-value less than 0.2 were included in the multivariate regression model. In order to find the optimal survival regression model, we used a backward elimination method which starts from a full model and eliminates highly non-significant variables in each step [1, 10].

Descriptive analyses were performed using Stata version 14.2 software. Other analyses such as draw plots, parametric and semi-parametric analyses were done using R packages (named “eha”, “SurvRegCensCov”, and “survival”) in R 3.4.1 software.

## Results

**Fig. 1:**
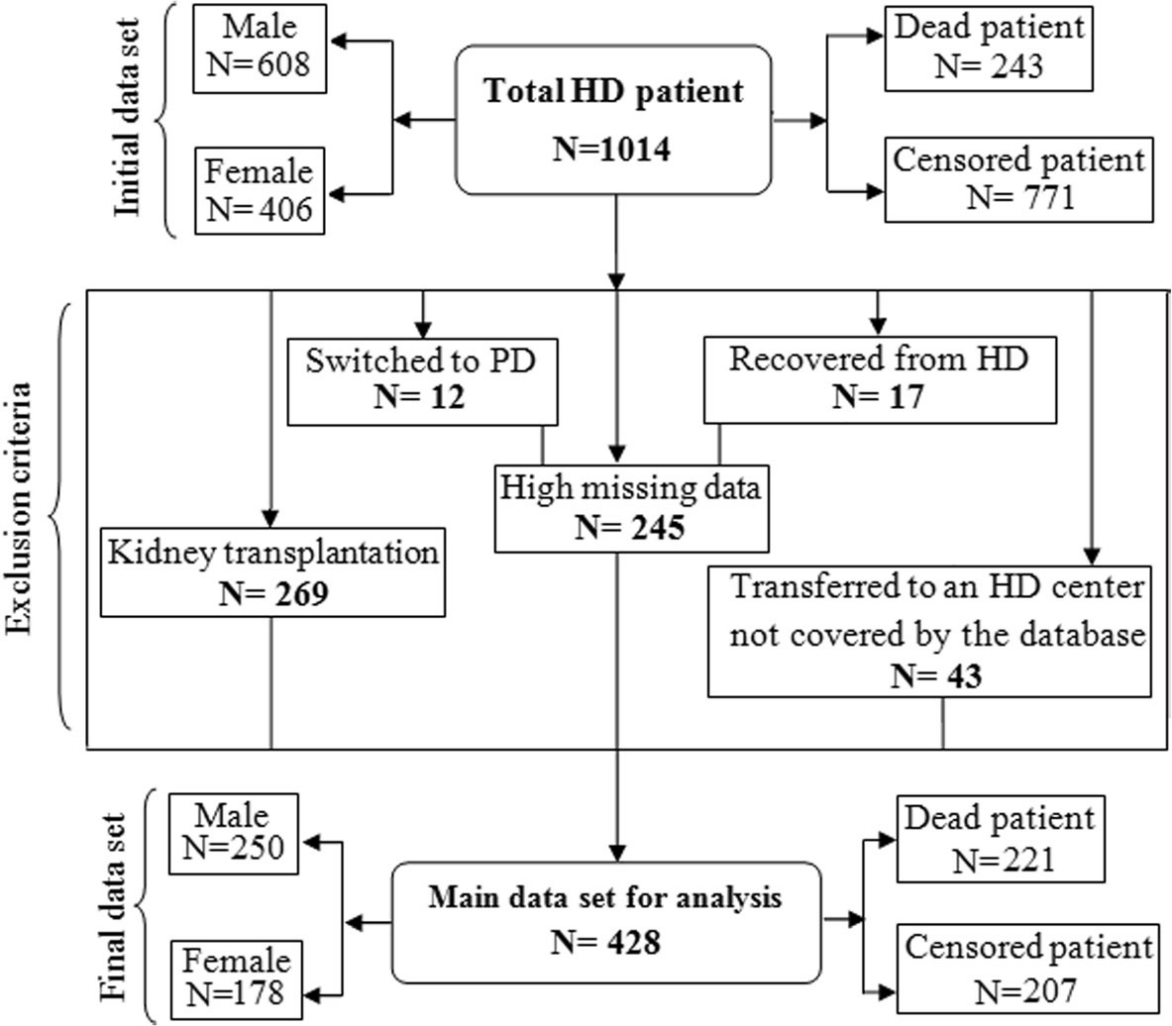
Structure of the ESRD patients’ data used in the analyses

Of the total of 1408 patients who were evaluated, 1014 who were on HD for at least 3 months were followed. After applying exclusion criteria, 428 patients were considered for the analysis (Fig. 1). With the mean ± SD age of 60 ± 16 years [range: 20–93 years] and BMI of 23 ± 4.6 Kg/m^2^ [range: 13–38 Kg/m^2^], they composed of 250 men (58%). The number of patients who experienced death was 221(52%); therefore, 48% of the survival time were censored (right censored). Table 1 demonstrates some of the patients’ demographic features and the measured laboratory values.

**Table 1:** Basic Demographic features and laboratory values of the study population

Demographic features	
Male sex (%)	250 (58.4)
Age (year)	60.0 (16.0)
BMI (kg/m^2^)	23.0 (4.6)
Laboratory Values	
WBC (10^3^/micl)	7.5 (4.5)
RBC (10^6^/micl)	3.8 (0.7)
Hemoglobin (g/dl)	10.2 (2.0)
HCT (%)	32.5 (5.0)
MCV (fl)	86.0 (7.5)
MCH (pg/cell)	27.0 (3.0)
MCHC (g/dl)	31.0 (2.0)
FBS (mg/dl)	131.0 (68.0)
Pre-dialysis BUN (mg/dl(	54.2 (18.0)
Post-dialysis BUN (mg/dl)	19.0 (8.0)
Pre-dialysis serum creatinine (mg/dl)	6.6 (3.0)
Post-dialysis serum creatinine (mg/dl)	2.9 (1.5)
Sodium (meq/lit)	139.5 (5.0)
Potassium (meq/lit)	5.0 (0.8)
Calcium (mg/dl)	8.7 (1.0)
Phosphate (mg/dl)	5.0 (1.5)
Uric acid (mg/dl)	6.6 (1.7)
PTH (pg/ml)	270.7 (237.0)
ALKPH (IU/L)	313.0 (186.0)
Total protein (mg/dl)	6.8 (0.8)
Albumin (g/dl)	3.8 (0.6)
Triglyceride (mg/dl)	127.0 (58.0)
Cholesterol (mg/dl)	153.0 (41.0)
LDL (mg/dl)	87.0 (30.5)
HDL (mg/dl)	37.7 (10.0)
Ferritin (μg/L)	319.0 (272.0)
Iron (μg/dl)	71.3 (53.5)
TIBC (μg/dl)	288.0 (94.0)
Transferrin Saturation (%)	25.0 (16.0)
Dialysis session indices	
Pre*-*dialysis weight *(Kg)*	64.0 (13.7)
Post*-*dialysis weight *(Kg)*	62.0 (13.5)
Pre-dialysis SBP (mmHg)	128.3 (19.2)
Pre-dialysis DBP (mmHg)	77.1 (10.1)
Pulse rate (per minute)	78.0 (6.0)
Kt/V_urea_	1.3 (0.3)
Average time of dialysis per week(min)	483.0 (177.0)
Ultrafiltration volume(ml)	2100.0 (1000.0)

Abbreviations: *ALKPH* alkaline phosphatase, *BMI* body mass index, *FBS* fasting blood sugar, *HCT* hematocrit, *HDL* high-density lipoprotein, *Kt/V_urea* adequacy of dialysis, *LDL* low-density lipoprotein, *MCH* mean cell hemoglobin concentration, *MCHC* mean corpuscular hemoglobin concentration, *MCV* mean cell volume, *PTH* parathyroid hormone, *RBC* red blood cell, *TIBC* total iron binding capacity, *UF* ultrafiltration volume, *WBC* white blood cell

The most common causes of ESRD were hypertension (31.5%), diabetes mellitus (24%), and simultaneous hypertension and diabetes mellitus (16.2%), while 15% of the ESRD patients had unknown etiology (Table 2).

**Table 2:** Underlying etiologies of ESRD in the study population

Underlying etiology of ESRD	No. (%)
Hypertension	135 (31.5)
Diabetes	103 (24)
Hypertension & Diabetes	69 (16.2)
Renal stone	21 (5)
Polycystic kidney disease	15 (3.5)
Glomerulonephritis	16 (3.8)
Others or unknown	69 (15)

*ESRD* End-stage renal disease

**Fig. 2:**
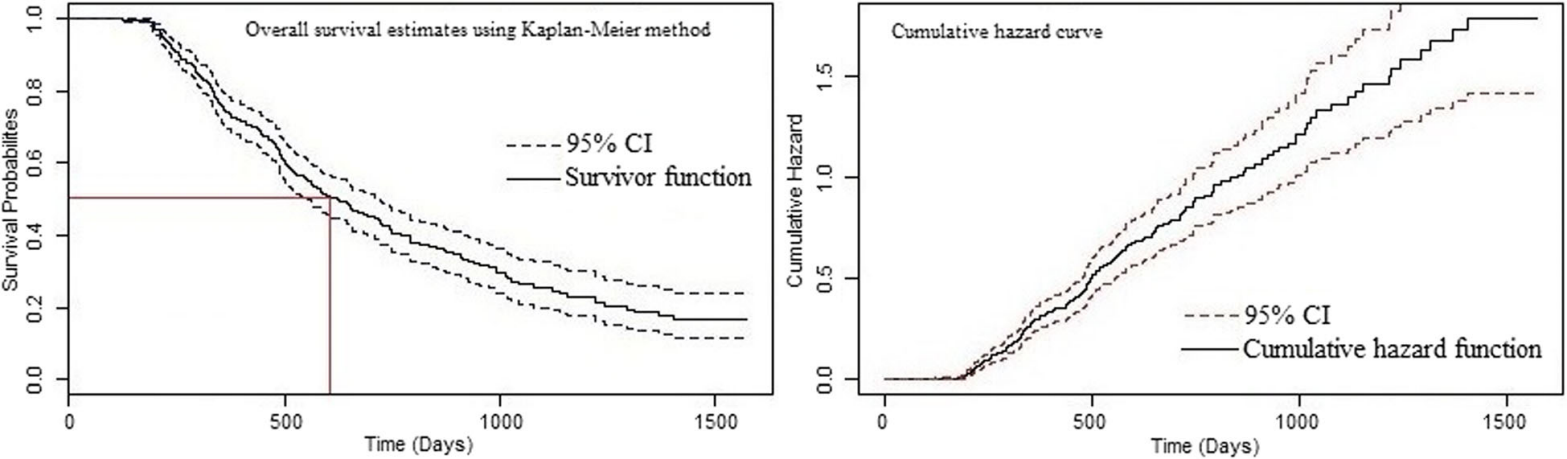
Kaplan-Meier and hazard curves for the study population (Median survival time equals to 624 days; 95% CI, 550 days to 716 days)

The estimates (95% CI) of the lower (25th), median (50th), and upper (75th) percentiles of the survival time were 357 (336 to 413), 624 (550 to 716), and 1120 days (991 to 1314), respectively. The median of the survival time was 624 days; it means that 50% of the subjects survived at least 624 days. Using the non-parametric KM method, the overall one-, two-, three- and four-year survival rates (95% CI) for the patients undergoing HD were calculated to be 74% (69 to 78%), 42% (37 to 48%), 25% (19 to 32%), and 17% (11 to 23%); respectively (Fig. 2, left).

There was no significant difference found between KM survival estimates of men and women (log-rank *p*-value = 0.971).

### Finding the optimal survival regression model as well as variables associated with survival

In order to find the best functional form of the survival time, the Cox PH model as well as four types of parametric survival methods including PH Gompertz, AFT (Weibull, lognormal, and log-logistic) models were performed.

The result of the multivariate analysis of the AFT log-normal model and the Cox-PH regression model, using Breslow manner, as well as HR (95%CI) of death and ETR (95% CI), which is the direct effect of a variable on survival time, are shown in Table 3. Our HD dataset did not demonstrate any violation of the PH assumption (all *p*-values> 0.05) and the *p*-value of the global test of the PH assumption was 0.473; as a results, we could apply the analysis of Cox-PH regression.

**Table 3:** The effect of the variables on the hazard of death and on the survival time of the study patients (expressed by event-time ratio) resulted from Cox proportional hazards and AFT log-normal; respectively

Cox PH regression			AFT lognormal regression	
Variable	HR(95% CI)	*P*-value	ETR(95% CI)	*P*-value
Age (year)	1.009 (0.999, 1.018)	0.075	0.997 (0.992, 1.002)	0.202
UF (ml)	1.146 (1.001, 1.313)	0.049*	0.951 (0.882, 1.025)	0.189
Sodium (meq/lit)	0.972 (0.943, 1.001)	0.061	1.013 (0.998, 1.028)	0.086
Calcium (mg/dl)	1.117 (0.984, 1.268)	0.086	0.961 (0.894, 1.033)	0.277
WBC(10^3^/ micl)	1.039 (1.015, 1.063)	0.001*	0.982 (0.968, 0.997)	0.018*
RBC (10^6^/ micl)	0.817 (0.671, 0.995)	0.044*	1.131 (1.017, 1.258)	0.023*
MCHC (g/dl)	0.887 (0.824, 0.954)	0.001*	1.067 (1.025, 1.109)	0.001*
Albumin (g/dl)	0.616 (0.483, 0.786)	< 0.001*	1.232 (1.080, 1.407)	0.002*
ALKPH (IU/L)	1.001 (0.999, 1.001)	0.144	0.999 (0.998, 1.0002)	0.243

*P-value < 0.05 is considered significant

*Abbreviations*: *coef* estimated coefficient, *CI* confidence interval, *ETR* event time ratio, *HR* hazard ratio, *ALKPH* alkaline phosphatase, *MCHC* mean corpuscular hemoglobin concentration, *RBC* red blood cell, *UF* ultrafiltration volume, *WBC* white blood

Cox-adjusted PH results indicated that the amount of ultrafiltration volume (UF) (HR = 1.146, P = 0.049), RBC count (HR = 0.817, *P* = 0.044), WBC count (HR = 1.039, *P* = 0.001), MCHC (HR = 0.887, *P* = 0.001), and serum albumin (HR = 0.616, *P* < 0.001) had significant effect on mortality.

While the results of AIC presented in the Table 4 revealed that in comparison with parametric models, the Cox-adjusted model had the weakest fit (with the highest AIC = 2258.52) and the AFT log-normal survival model (with the lowest AIC = 665.06) had the best overall fit.

**Table 4:** Assessment of the quality of the fitted event-time models using AIC criterion

Parametric Model	Log likelihood (Null)	Log likelihood (Model)	AIC Criterion
Cox PH model	− 1145.77	−1120.26	2258.52
PH Gompertz	− 396.56	− 370.91	763.82
AFT weibull	−370.28	− 341.46	704.92
AFT Lognormal	− 343.49	− 321.53	**665.06**
AFT Loglogistic	− 350.85	− 327.06	676.13

*Abbreviations*: *AIC* Akaike Information Criterion, *AFT* accelerated failure time, *PH* proportional hazard

**Fig. 3:**
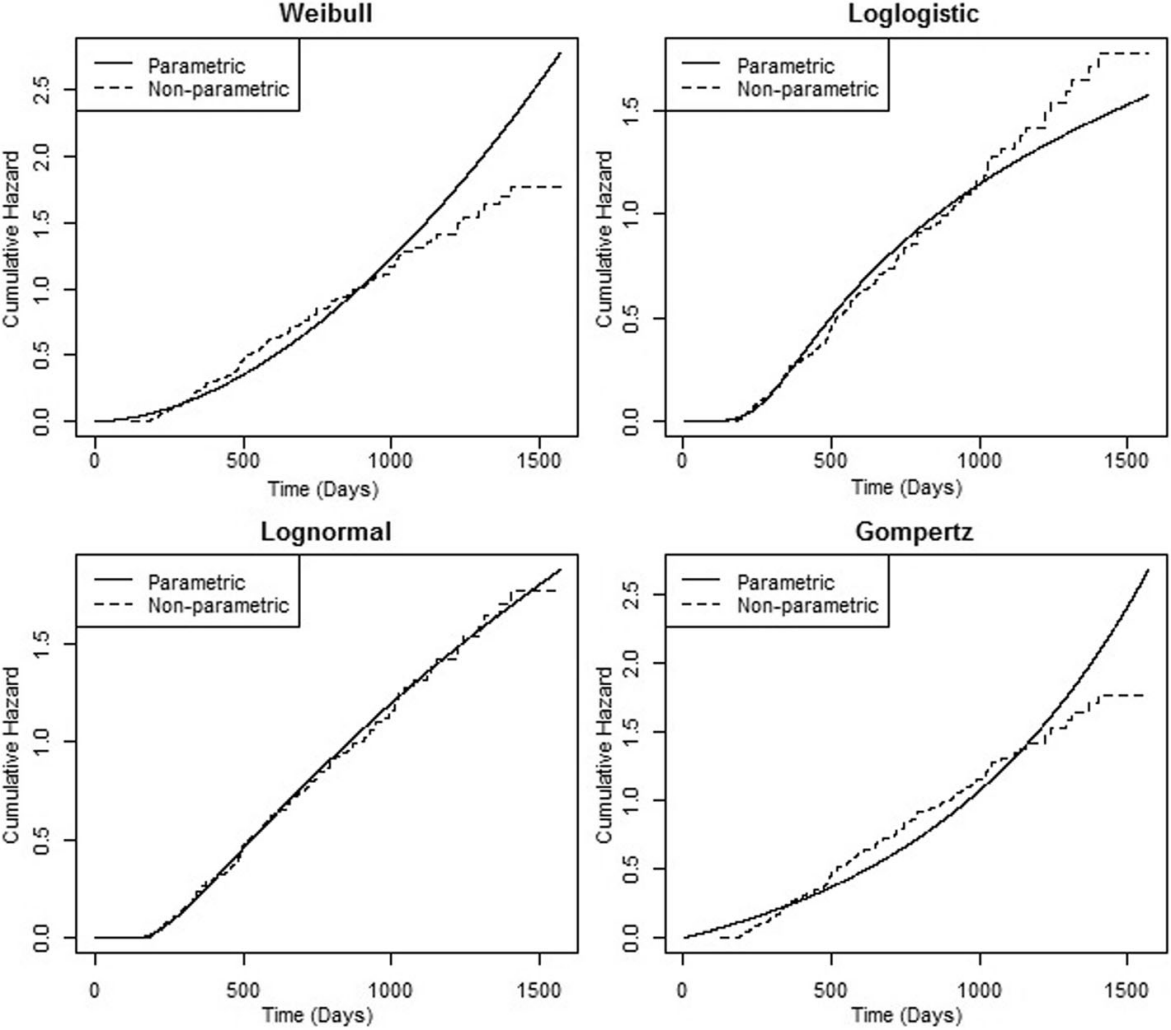
Graphical evaluation of various fitted models via comparison between non-parametric and parametric methods

**Fig. 4:**
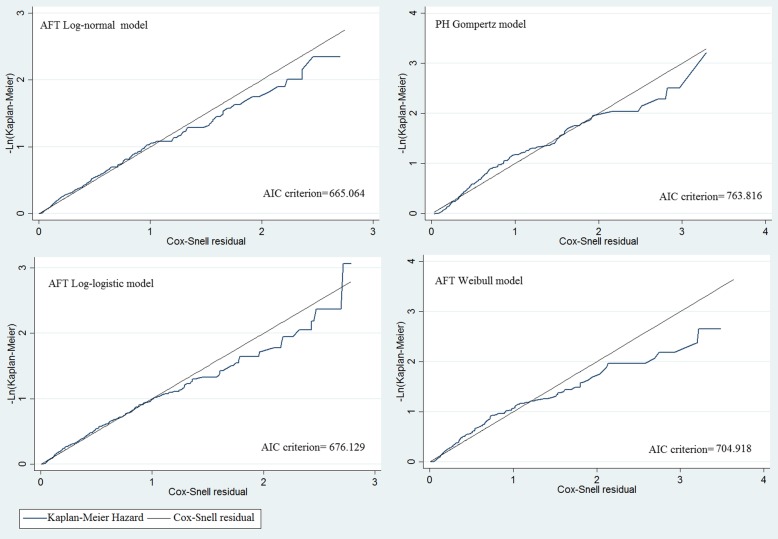
Graphical evaluation of various fitted models via comparison between non-parametric Kaplan-Meier hazard and the Cox–Snell residuals

As an alternative, we used a graphical comparison between non-parametric and parametric methods in order to check the adequacy of the AFT log-normal model (Fig. 3). As seen in Fig. 3, it seems that the AFT lognormal model fits well to the non-parametric model and can be applied appropriately for analyzing the survival time. Also, as indicated in the Fig. 4, the log-normal model fits the HD data best. Therefore, we used the AFT log-normal to determine associated factors affecting survival time of HD patients.

The result of AFT log-normal analysis indicated that blood WBC count, RBC count, MCHC, and serum albumin had significant effects on survival time of HD patients (all *p*-values< 0.05).

As it is shown in Table 3,which is the results of the AFT log-normal model, the ETR (95% CI) of WBC count was 0.982(0.968 to 0.997).This means that the overall survival time of the HD patients decreases approximately about 2% for each 10^3^ per microliter increment in the WBC count (*p*-value = 0.018). The ETR of RBC in association with overall survival demonstrated that the survival time of patients undergoing HD significantly increased (by 13%) for each 10^6^ per microliter increment in RBC count [ETR (95%CI) =1.131(1.017 to 1.258), *P* = 0.023].

The ETR (95% CI) of 1.067(1.025 to 1.109) for MCHC represents that rise in MCHC for each unit (in g/dl) was associated with almost 7% increase in the survival time (*P* = 0.001). Our findings also revealed that for each unit (in g/dl) increase in the serum albumin, the survival time for HD patients increased by an approximately 23% [ETR (95%CI) =1.232(1.080 to 1.407), *P* = 0.002].

It was interesting to note that the serum sodium concentration were marginally significant in the AFT log-normal model (*P* = 0.086) while age, ultrafiltration volume, serum calcium, and ALKPH were not significant (*P* = 0.202, 0.189, 0.227, and 0.243 respectively).

## Discussion

The population of patients suffering from ESRD is growing worldwide; consequently, more attention is being paid to the lifespan of ESRD patients and the factors associated with survival.

Because of the familiarity of the medical researchers with the Cox-PH model, most of the literature uses this type of survival analysis. There is no need to know the distribution of survival time [11], but establishing proportionality (PH) assumption is necessary [1]. A systematic review demonstrated that merely 5 % of the international journals have considered this important assumption in their analyses [3]. In spite of the long history of the parametric event-time models, they have largely been ignored in the medical literature.

Before going through the Cox-adjusted PH analysis, we initially made sure that PH assumption for each factor, and also in general, was established. The results of AIC criteria stated that with respect to the parametric methods, the Cox-adjusted PH model had the weakest efficiency and the parametric AFT log-normal event-time model seems to have good accuracy. This was also confirmed by graphical methods and Cox-Snell residuals (Figs. 3 and 4).

Recently, several studies have investigated the efficiency of the semi-parametric Cox-adjusted PH and parametric event-time models in survival analysis of a variety of diseases [5, 11, 12]. A study done on gastric cancer patients reported that the AFT log-normal model could be replaced with Cox-PH model in the survival analysis [5]. Orbe et al. performed a simulation study and reached the same conclusion [11]. Findings from a study conducted by Wang et al. showed that parametric log-normal survival model was the best functional form of the age at the time of diagnosis of gallbladder cancer while the Cox-PH method performed poorly [12]. To the best of our knowledge, there was no study about the role of parametric survival models in HD patients.

Multivariate analysis of the AFT log-normal model indicated that the increased level of RBC, MCHC, and serum albumin were associated with increase in the survival time for HD patients. In contrast, as level of WBC counts increases, survival time of patients undergoing HD declines. For each unit rise in WBC count (in 10^3^/micl), the survival time of HD patients decreases about 2%. A study done on HD patients in Taiwan showed that total WBC count predicts one-year all cause and cardiovascular mortality [13]. Another study which was done in the United States on a large population of HD patients concluded that an increased neutrophil count and reduced lymphocyte count are risk factors for mortality [14]. Therefore, due to its potential impact on the survival time as one of the major elements of the complete blood count, the WBC count must be considered in the laboratory evaluation of HD patients. However, because there was no data available on the differential WBC count, we were not able to consider the neutrophil or lymphocyte count in the analysis.

To the best of our knowledge, our study is the first one which has proven that RBC count is an independent predictor of survival in HD patients. Anemia and low hemoglobin levels are well known indicators of lower survival in HD patients. However, no study found that RBC count is a mortality index independent of Hb level. We found that for each 10^6^ per microliter rise in RBC count, patients’ survival time increases by 13%.

Higher MCHC level, which reflects the concentration of hemoglobin in a red blood cell, is another factor that significantly contributes to the increased survival time. According to the vast literature review of ours, there was only one study on patients with ESRD which indicated that low MCHC is associated with lower short term survival [15]. Tennankore et al. found that macrocytosis is associated with mortality in stable HD patients [16]. Iron deficiency, which can lead to low MCHC level, could account for the lower survival time of patients with low MCHC. However, it is interesting that lower MCHC was associated with lower survival time independent of serum ferritin level and transferrin saturation. This result emphasizes the role of this important index as one of the items which could have remarkable impact on survival of HD patients and should be highlighted as an important item in the laboratory evaluation of HD patients, besides iron status parameters.

Serum albumin is an independent and powerful prognostic index for HD patients. Current findings is consistent with previous studies [17, 18] which demonstrates that as the serum albumin increases, the probability of survival as well as survival time increases. We found that for each g/dl increment in serum albumin, the HD patients’ survival time increases by an approximated 23%. We believe that this is the first study which has brought at the amount of change in survival time of HD patients according to the changes in the laboratory values, which were found to be independent predictors of survival.

One the major advantages of this present study is the large number of the study population and a good follow-up time. Besides, we included several variables in the analysis including laboratory values and HD sessions associated variables.

There are some limitations to our study. We were not able to consider the cause of mortality because it was not available in the dataset. Besides, the type of vascular access was not considered in the analysis, the variable which could have some impact on the survival. Inflammatory markers, which could modify the relationship of some of the variables with survival, are the other variables which were absent in our study.

## Conclusion

In conclusion, our study showed that parametric survival models might be more appropriate than semi-parametric Cox PH models for analyzing survival of HD patients. By considering the potential factors influencing survival, parametric models can estimate not only the hazards of death but also the change in the survival time of HD patients. Among the parametric models, AFT log-normal had the best fit to our HD patients’ data. Total WBC count, RBC count, MCHC and serum albumin were found to influence survival time of HD patients.

## Abbreviations

AFT: Accelerated failure time
AIC: Akaike’s information criteria
ALKP: Alkaline phosphatase
BMI: Body mass index
BUN: Blood urea nitrogen
DBP: Diastolic blood pressure
ESRD: End-stage renal disease
ETR: Event time ratio
FBS: Serum concentrations of fasting blood sugar
Hb: Serum hemoglobin concentration
HCT: Hematocrit
HD: Hemodialysis
HDL: High-density lipoprotein
HR: Hazard ratio
KM: Kaplan-Meier
LDL: Low density lipoprotein
MCH: Mean cell hemoglobin concentration
MCHC: Mean corpuscular hemoglobin concentration
MCV: Mean cell volume
PD: Peritoneal dialysis
PH: Proportional hazards
PTH: Parathyroid hormone
RBC: Red blood cell count
RRT: Renal replacement therapy
SBP: Systolic blood pressure
SUMS: Shiraz University of Medical Sciences
TIBC: Total iron binding capacity
WBC: White blood cell count
